# Non-readmission decisions in the intensive care unit: A qualitative study of physicians’ experience in a multicentre French study

**DOI:** 10.1371/journal.pone.0244919

**Published:** 2021-01-14

**Authors:** Marine Jacquier, Nicolas Meunier-Beillard, Fiona Ecarnot, Audrey Large, François Aptel, Marie Labruyère, Auguste Dargent, Pascal Andreu, Jean-Baptiste Roudaut, Jean-Philippe Rigaud, Jean-Pierre Quenot

**Affiliations:** 1 Department of Intensive Care, François Mitterrand University Hospital, Dijon, France; 2 Lipness Team, INSERM Research Centre LNC-UMR1231 and LabEx LipSTIC, University of Burgundy, Dijon, France; 3 INSERM CIC 1432, Clinical Epidemiology, University of Burgundy, Dijon, France; 4 DRCI, USMR, Francois Mitterrand University Hospital, Dijon, France; 5 EA3920, Department of Cardiology, University Hospital Besancon, Besancon, France; 6 Department of Intensive Care, Dieppe General Hospital, Dieppe, France; 7 Espace de Réflexion Éthique de Normandie, University Hospital Caen, Caen, France; 8 Espace de Réflexion Éthique de Bourgogne Franche-Comté, Dijon, France; Institute of Mental Health, SINGAPORE

## Abstract

**Purpose:**

Deciding not to re-admit a patient to the intensive care unit (ICU) poses an ethical dilemma for ICU physicians. We aimed to describe and understand the attitudes and perceptions of ICU physicians regarding non-readmission of patients to the ICU.

**Materials and methods:**

Multicenter, qualitative study using semi-directed interviews between January and May 2019. All medical staff working full-time in the ICU of five participating centres (two academic and three general, non-academic hospitals) were invited to participate. Participants were asked to describe how they experienced non-readmission decisions in the ICU, and to expand on the manner in which the decision was made, but also on the traceability and timing of the decision. Interviews were recorded, transcribed and analyzed using textual content analysis.

**Results:**

In total, 22 physicians participated. Interviews lasted on average 26±7 minutes. There were 14 men and 8 women, average age was 35±9 years, and average length of ICU experience was 7±5 years. The majority of respondents said that they regretted that the question of non-readmission was not addressed before the initial ICU admission. They acknowledged that the ICU stay did lead to more thorough contemplation of the overall goals of care. Multidisciplinary team meetings could help to anticipate the question of readmission within the patient’s care pathway. Participants reported that there is a culture of collegial decision-making in the ICU, although the involvement of patients, families and other healthcare professionals in this process is not systematic. The timing and traceability of non-readmission decisions are heterogeneous.

**Conclusions:**

Non-readmission decisions are a major issue that raises ethical questions surrounding the fact that there is no discussion of the patient’s goals of care in advance. Better anticipation, and better communication with the patients, families and other healthcare providers are suggested as areas that could be targeted for improvement.

## Introduction

The criteria for admitting a patient (or not) to the intensive care unit (ICU) have been widely discussed in the literature [1, 2]. Conversely, the literature is sparse concerning decisions not to re-admit patients to the ICU within a single hospital stay, after they had initially been discharged from the ICU to a lower-acuity ward. Such decisions are made in only 6 to 11% of patients who are discharged from the ICU [3–5]. Yet, whether re-admission would be appropriate should be questioned more often for patients for whom management in the ICU was difficult, in those whose vital or functional prognosis is severely impaired, or for patients with a heavy burden of comorbidities.

The decision not to readmit a patient to the ICU is founded on the ICU physicians’ conviction that a new admission to the ICU will not yield any further benefit for the patient in terms of survival and/or future quality of life. Consequently, providing life support therapies to the patient in the ICU that would serve no purpose other than artificially maintaining life, and would represent a situation of “unreasonable therapeutic obstinacy” [6]. In such a case, it becomes mandatory to re-orient the patient towards more appropriate pathways, which can provide support and accompaniment (e.g. palliative care) adapted to the patient’s needs and aligned with the desires of both the patient and their family.

The latest French legislation on the rights of patients at the end of life dates from 2 February 2016 (the so-called Claeys-Léonetti law) [7], and supersedes the previous versions dating from 2002 [8] and 2005 [9]. The 2016 law enshrines the concept of the “collegial” (or shared) decision regarding limitation and/or withdrawal of treatment in patients who are unable to express themselves (e.g. due to severity of disease). Collegial (or collegiate) decisions are made collectively, in a multidisciplinary meeting bringing together physicians and other stakeholders (nurses, family, ethics consultants, other specialists…). The decision can be made during the hospital stay, in order to avoid over-treating the patient (i.e. “unreasonable therapeutic obstinacy”, as per the term used in the French legislation) [6, 10–12], or at the end of the hospital stay, at which time it should be noted in the patient’s medical file that he/she will not be admitted to the ICU again at any time in the future.

It is of paramount importance that the patient and their family be adequately informed, that the patient’s wishes be taken into account, and that a collegial decision-making process be implemented for decisions not to re-admit to the ICU [13].

A recent survey of practices [14] among physicians from 58 ICUs in France reported that the decision not to re-admit a patient to the ICU was most frequently made at the end of the ICU stay (in 87%), using a collegial decision-making process (89% of cases), and 93% of the 165 respondents indicated that the decision was recorded in the patient’s medical file. However, the patient’s family were informed in only 73% of cases, and more alarmingly, only 29% of respondents stated that they informed the patient. Further, the physicians in the ward that was to receive the patient after discharge from the ICU were informed or involved in the decision in less than 30% of cases [14].

Clearly, the question of non-readmission poses an ethical dilemma for ICU physicians. For a given patient, re-admission to the ICU can be seen as unreasonable therapeutic obstinacy by some, but as a loss of opportunity by others. Therapeutic obstinacy refers to any curative strategy that is futile and unsubstantiated by the patient’s prognosis in terms of survival and quality of life, or that is not aligned with the patient’s wishes, expressed either directly or through a surrogate or written document (e.g. advance directives). On condition that a collegial decision-making procedure is scrupulously respected, French law allows physicians not to initiate, or not to pursue a curative strategy (i.e. life-support) if it is deemed to be futile and unjustified given the patient’s prognosis in terms of survival or quality of life. The law underscores the physician’s obligation to initiate palliative management if the patient is not going to receive life-prolonging therapy, so that the patient may experience a peaceful end-of-life.

Against this background, we decided to build on our previous investigations in this area showing that practices vary, by performing a qualitative study among ICU physicians, using semi-directed interviews. The objective of the study was to describe and understand the attitudes and perceptions of ICU physicians regarding the question of non-readmission of a patient to the ICU.

## Methods

We performed a multicenter, qualitative study using semi-directed interviews. All the qualified physicians working full-time in the ICU of the five participating centres (two academic teaching hospitals and three general, non-academic hospitals) at the time of the study were invited to participate by personal invitation. Trainees or medical students were not eligible for participation. Interviews were performed between January and May 2019 by a qualitative researcher (MJ) and sociologist with experience in clinical research and ICU care (NMB).

We developed an *ad hoc* guide to prepare the interviews using a methodology previously described elsewhere [13, 15, 16]. Regarding the interview guide, participants were asked to describe how they experienced non-readmission decisions in the ICU. They were invited to expand on the manner in which the decision was made, but also on the traceability (i.e. whether the decision was noted in the medical file and whether downstream care units and/or the family were informed) and timing of the decision. The interview guide is detailed in Table 1. The original French version is provided in S1 Table.

**Table 1:** Interview guide.

1. Was admission to the ICU anticipated? Was it appropriate in your view?
2. Do you enquire about advance directives?
3. Can you tell me about how a decision not to readmit is made in your practice?
4. Could you describe the profile of patients that are not to be re-admitted to the ICU?
5. Is there a role for the palliative care team?
6. Are non-readmission decisions ever reversed in your experience?

As with all qualitative interviews, the questions were open ended and intended as a prompt to get the respondent to talk about the aspects that were most important to them, and to voice these concerns in their own words. Interviews were performed in a dedicated medical office in each center. No other persons were present during the interview.

All interviews were recorded and transcribed in their entirety for later analysis. Data were encoded to guarantee the anonymity of the participants. The corpus of discourse from all the interviews with physicians was analyzed using textual content analysis as previously described by our group elsewhere [13–15]. In brief, interviews were coded independently by 2 of the coauthors (MJ, NMB), the aim being to identify and categorize the different themes occurring in a cross-sectional manner across all interviews (i.e. topics common to several individuals). Each theme is then considered as a meaningful independent unit of discursive language. The different themes that arise during the interviews are recorded, taking into account major themes (significant points that are of major importance and well developed by the participants) and secondary themes (less well developed by the participants). The first level of analysis was performed individually by each researcher, then meetings were held to harmonize and decide on the major and secondary themes to be retained, and their regrouping into subject categories. Differences in interpretation were resolved by discussion and consensus.

Interviews were conducted until saturation was reached (i.e. the point at which new interviews failed to bring forth any new elements on any of the points in the interview grid) [17].

The study was approved for the all participating centers by the ethics committee (Comité de Protection des Personnes Est I) of the University Hospital of Dijon, France at its meeting held on December 13, 2018. Informed consent was implied by the fact that all participants volunteered to be interviewed. Participants were made aware that quotes from their interviews might be used in scientific publications to substantiate the discussion.

## Results

All the physicians invited in the 5 ICUs participated in the study (n = 22). Interviews lasted on average 26 ± 7 minutes. There were 14 men and 8 women, and the average age of participants was 35±9 years. The average length of ICU experience was 7±5 years. Physicians with more than 2 years’ experience in the ICU accounted for three quarters of those interviewed.

### In your view, was admission to the ICU appropriate and/or anticipated?

The majority of respondents said that they regretted the fact that the question of non-readmission was not addressed before the initial admission to the ICU, although they acknowledged that the ICU stay did lead to a more thorough contemplation about the overall goals of care. The idea of multidisciplinary team meetings could help to anticipate the question of readmission within the patient’s healthcare pathway.

“*I’m not saying this is the case for everyone*, *but eh… maybe they [the patients] should never have been there the first time and so the situation wasn’t really anticipated”*

“*If you address the question beforehand*, *it’s always easier when things are calm and unhurried*, *and in the end*, *the decisions are better informed than when you decide not to readmit to the ICU”*

“*So it’s best to try to discuss it in advance*, *but that’s not often the case with the referring physicians who are dealing with the patients”*

“*In oncology*, *they have multidisciplinary meetings about management*, *but the ICU physicians are never involved*, *although we know well that at some point*, *the patient will deteriorate and then the question is*, *how far do we go*?*”*

Regarding advance directives, the physicians responded that they check if there are any, but all were unanimous in stating that they are extremely rare, and do not provide sufficiently detailed information about the patient’s wishes for end-of-life care.

“*We ask if there are any*, *but it’s very rare*, *even in nursing homes…*. *They never have AD*. *It’s happened maybe once [that someone had AD]”*.

“*When they have AD*, *they’re always the same–“don’t go overboard”… [respondent adopts a skeptical tone] nobody wants that*. *So I think that they’re not very useful*, *although at least it does mean that the person has given some thought to their end-of-life*, *that’s a good thing”*.

“*For the time being*, *we’re not there yet*. *If we prepared advance directives that were associated with a healthcare plan*, *like advance care planning*, *then in that case*, *it would be done a long time in advance”*.

### The decision not to readmit: Intended as a collegial process

All the participants highlighted the fact that in the ICU, since the updated 2016 legislation, there is now a culture of collegial decision-making, although the involvement of the patients, the families and other healthcare professionals (including general practitioners and others specialists) is not systematic. Conversely, the timing and traceability of the decisions not to readmit are heterogeneous, according to the physicians interviewed.

“*Well*, *here*, *often when it’s a case of not readmitting*, *we don’t necessarily have a formal meeting*, *unless it’s a young patient or a really complicated case”*

“*It’s relatively formal*, *we frequently have meetings about the therapeutic orientation and I’d say about 40% of patients are contra-indicated for readmission”*.

“*Above all*, *I’ve noticed that we’re not alone any more*. *We analyse*, *we write it down*, *we consult others*, *and that’s really helpful”*.

“*For decisions not to readmit*, *I think it’s good that it’s the ICU physicians who decide*, *but that doesn’t prevent us from asking others for their opinion”*.

“*I estimate that we always leave about 7 to 10 days*, *to think about it*, *before making any decisions”*.

“*Maybe we need to give more details about why we made that decision”*.

The role of the patient’s other treating physicians depended largely on their specialty:

“*There’s some specialists that we work with more easily than others*. *Some of them will actually come up to our department to discuss a case*, *and give us a few pointers…”*

“*The GP should have a role to play [respondent raises eyes to heaven] but to be honest*, *we rarely involve them in this kind of decision*. *The GP is very pragmatic*, *and often repeats what the patient said to him*, *and then most often*, *the family confirms that”*

The position of the caregiving team (nurses, nurses’ aides) is variable, even though they are in closest contact with the patient.

“*[The nurses] hear things*, *they come to the meetings*, *but they don’t decide*. *In any case*, *we*, *I mean the physicians*, *maybe it’s true that we don’t give them much chance to speak out”*

“*In general*, *I think they prefer not to come down on one side or the other*, *but they are in closer contact with the patients*, *and they can see the suffering that our treatment is inflicting*, *so they’re on the front line*. *So I think it’s justified that we ask the nurses’ opinion*, *they do provide a new perspective”*

“*If it’s a decision that’s made at the daily staff meeting at lunchtime and the patient is discharged in the afternoon*, *then I don’t think the paramedical staff are necessarily informed about the non-readmission”*

The physicians seek to avoid a situation where physicians in downstream care units have to make the decision about whether or not to send the patient back to the ICU, because the downstream physicians do not always know the patient’s full history, they have not followed the patient since the beginning, and may also be unqualified to judge whether the therapies available in the ICU are likely to be of any real benefit to the patient.

“*The aim is to avoid a situation where the providers in the downstream unit after the ICU have to make that decision*, *but that doesn’t mean they’re not involved in the decision”*

The place of the patient and family seems difficult to ascertain in this decision-making process, because one has to be kind, and find the “right words”, and that can require a certain amount of experience. Sometimes decisions pertaining to re-admission (or non-readmission) may be made by the medical team, taking account of the opinions expressed by the family. However, the family may not necessarily be present when the decision is made, and they may not necessarily be informed about the decision either, if the medical team judges that the information would do them more harm than good.

“*In practice*, *I find that we rarely discuss things with the patient…”*

“*Maybe I wasn’t clear enough when I said “we won’t take you back*, *in the ICU”*, *but I find it’s a very negative way of saying it”*

“*The decision not to re-admit is hard to explain to patients because you kind of have the impression that you’re sentencing them*!*”*

“*It’s more a question of us looking for information from the family*, *rather than involving them directly in the decision*, *when the decision is medical”*.

“*It varies really*. *There are families that understand well*, *who are perfectly clear about the different reasons*. *But there are others*, *you tell them exactly the same thing*, *and you realize that they didn’t really understand what you were trying to tell them…”*

### What kind of patients are not to be re-admitted to the ICU?

Age, the presence of comorbidities, the acute and underlying pathology and its potential for progression, autonomy and quality of life are all criteria that the ICU physicians take into account when deciding whether or not to admit a patient to the ICU. However, when we address the question of non-readmission, the patient’s prior healthcare trajectory in the ICU is mentioned by the participants.

“*Age comes into it a little*, *but it’s not the most important thing”*

“*In our decision*, *we take into account an incurable disease that’s going to progress quickly*, *and that we have no treatment for”*

“*Most of the time*, *we try to base our decision on the patient’s autonomy at home”*

“*I rarely ever know the degree of autonomy*, *I don’t take the time to calculate it*, *and I don’t know enough about it*, *but we take into account the activities of daily living”*

“*If we went so far as to put an ECMO in place*, *then we won’t give up so easily*, *and we’ll keep going*, *but we’d probably be more unreasonable with a readmission”*.

### The role of the palliative care team

Although the palliative care team was recognized as aiding communication with the patient and family, most physicians in our study did not know how or when to involve palliative care and lacked a formal mechanism for making that involvement routine.

“*I don’t know if it’s our job to do that*, *I don’t really know… I’ve never gotten the palliative care team involved”*

“*In general*, *they have an outsider’s view*, *they manage to find other ways of saying things*, *and that can help us clarify things a bit…*.*”*

### The non-readmission decision is not set in stone

Most of the time, the decision not to re-admit a patient to the ICU can be reversed, according to the participants in our study.

“*There may be a doubt at a given time*, *but it’s not set in stone*. *If some day*, *a doctor sees the letter at 3 o’clock in the morning*, *I don’t mind discussing it again*, *but that hardly ever happens”*

“*Not taking back a patient who’s 92 years old*, *with a flu complicated by acute respiratory distress syndrome*, *that’s not the same thing as not taking them because they have a urinary tract infection that can be treated with simple antibiotics*. *There’s the context*, *but there’s also the reasons why they’re asking us to take the patient back”*

Economic aspects, and the ICU’s admission policy may also enter into play in the decision not to re-admit a patient.

“*It depends on the context… whether we’re under pressure or not*. *Definitely*, *if it’s a time when we have lots of beds free*, *then admissions and re-admissions will be more liberal”*.

## Discussion

To the best of our knowledge, this is the first qualitative study using semi-directed interviews to investigate ICU physicians’ perceptions on the issue of non-readmission of patients to the ICU. It provides an insightful complement to a previous study using a questionnaire that explored the quantitative aspects of this same question [14]. The present study confirms that non-readmission decisions are a major issue, as suggested by our previous work. Several main themes are expressed by the physicians, which raise ethical questions surrounding the fact that there is no discussion of the patient’s goals of care in advance, particularly the question of potential admission to the ICU. The participants also mentioned the difficulties they experienced in including all those involved in the patient’s care, as well as difficulties with communication about a non-readmission decision. Finally, they emphasized the patient profile for whom the question of non-readmission to the ICU should probably be addressed more frequently. In this regard, communication with other healthcare providers about medical decisions is of a different nature to the communication with the patient/family. The medical features will be discussed with other healthcare providers (downstream units, GPs etc), and a decision not to readmit may be made, based on the patient’s progress, likely outcome in the short and long term, and the utility of a future re-admission to the ICU in a context of scarce resources. The physicians in this case are navigating a fine line between loss of opportunity for a patient who might potentially benefit from ICU care, and “unreasonable obstinacy”, i.e. providing futile care to a patient who will not benefit and will by the same token, deprive someone else of getting that care.

The majority of physicians who participated in the present study felt that improvements to the decision-making process for non-readmission to the ICU could focus on two main areas. Firstly, improved anticipation, by discussing the possibility of ICU admission with other healthcare providers when developing the patient’s goals of care. For example, this could take the form of formal multidisciplinary meetings involving ICU physicians as well as referring physicians, specialists and GPs. Secondly, as a corollary to the first area, the continued involvement of the patients, family and healthcare providers in the decision-making process, across the whole spectrum of care, whether in the hospital or not. It should be noted here that the in the French model, the final decision is made by the physician in charge of the patient, and the involvement of the patient/family consists in expressing their preferences and making their values known.

In light of literature data and daily practice, we note that in ICUs, physicians and paramedical staff often wonder about whether the decisions being made for a patient are appropriate during an ICU stay [18]. Above all, the ethical principles of not doing harm and acting in the patient’s best interest should prevail. Even without considering the high cost of an ICU stay, delivering care that is perceived as unjustified or inappropriate can create distress, especially if the care is found to be futile [19]. The potentially inappropriate nature of treatment in intensive care has recently been addressed by guidelines from several professional societies, in the aim of inciting the medical profession to lead public engagement efforts and advocate for policies and legislation about when life-prolonging technologies should not be used [20]. Potentially inappropriate care can cause physical, emotional, spiritual and financial distress for patients, their family and society [21]. Similarly, as mentioned by the physicians interviewed in the present study, daily meetings between the medical providers should be an integral part of good practice [22] and could be associated with a decline in conflicts and in provider suffering [23, 24]. By allowing a shared decision to be made between several providers, a more balanced and less subjective assessment of the appropriateness of ICU admission might be achieved. This will meet the need for more consistent processes among the medical providers to consider the patient’s trajectory/prognosis and assess the utility of a future re-admission. This process must include all relevant clinicians, such as the GP, the palliative care team, the nursing staff. In parallel, there is also a need to meaningfully involve the patient/family in the decision-making process, by eliciting their views and preferences, and taking these into account. Of note, the final decision remains up to the physician in charge of the patient. Then, the final link in the chain is the need for transparent communication with the family about how non-readmission decisions are made and the ultimate decision made. Patients and their families should also be encouraged to think about what comes after the ICU, and to anticipate complications and sequelae that may impact on the future physical, emotional, cognitive or even financial state, not only of the patient but also of the patient’s family [25–29].

A recent prospective, multicentre centre study in France that included 1075 patients [30] found that 14.4% of admissions to the ICU were felt to be inappropriate by the ICU physicians. The fact that ICU care or an ICU stay could be considered inappropriate should prompt us to think about ways to limit, or even avoid such situations, which can have nothing but negative consequences for the patient, the family and the healthcare professionals. A team from Brazil [31] recently reported in a prospective, before-and-after study, that implementation of a decision-aid tool for ICU triage was associated with a reduction in potentially inappropriate ICU admissions. Other strategies, suggested by the physicians interviewed in our study, such as advance care planning, could also help to address the role of the ICU in the patient’s goals of care, while consultations with ethics or palliative care teams could be another useful option [32–36]. Clearly, all the players need to be involved and the ICU physicians should remain the preferred intermediary for discussing decisions about whether to admit a patient or not to the ICU, and all the more so when deciding whether to re-admit a patient or not [14, 37–40]. Several arguments support this position. The ICU physician has expertise in life-support therapy, and can also evaluate the prognosis based on the existence and/or treatment of organ failure. Finally, the ICU physician is also qualified to explain to the patients and family the indications, limits and consequences of intensive care [41].

In our study, the physicians interviewed suggest that a collegial and multidisciplinary approach would be of significant value, in particular formalized meetings to decide specifically on non-readmission to the ICU [11, 14, 39, 42]. Implementing daily collegial reflection for each patient about their goals of care has been shown to lead to shorter times to decision, and improved palliative care management [43]. This in turn was shown to reduce the frequency of burn-out among the caregiving staff [12]. However, it should be noted that collegial discussions may influence the decision-making process because of the emotional dimension [10]. Furthermore, nursing staff are often more realistic than the physicians with regard to a clinical situation, especially as regards loss of autonomy for the patient. In any case, it is the physician’s duty to provide clear, transparent and honest information to the patient and family [44]. Nonetheless, it should be noted that informing a patient who is in a situation of vulnerability, even about the fact that non-readmission to the ICU was discussed, can be particularly impactful. It would be not be justified, on the pretext of transparency, to show a complete lack of beneficence, especially since the patient also has the right not to know [8]. Therefore, the physician has to impart information with a certain degree of circumspection. Indeed, the patient has the right NOT to receive information pertaining to their own health, if they prefer not to. Some people do not like to hear bad news, and prefer not to ask questions. People who are weak, frail and/or vulnerable, or worried relatives of ICU patients, may not be receptive to discussions about the goals of care at that particular time. Nevertheless, the family cannot be left out of the decision-making process in the ICU, especially for non-readmission decisions [45, 46]. It has also been shown that an intensive communication strategy in end-of-life situations in the ICU can help to reduce not only the symptoms of depression in the family at 6 months, but also the duration and cost of ICU care for the deceased patients [47]. Undoubtedly, during discussions with the family, the patient’s age, comorbidities, autonomy, former quality of life or expected quality of life after the ICU, and more generally, the patient’s life trajectory should all be taken into consideration [48, 49].

There clearly exists a dilemma in the French healthcare system, namely the flagrant gap between what the law stipulates regarding decision-making practices, and what actually happens in practice. The paternalistic model has not yet disappeared altogether, and although French physicians work within a decision-making model that affirms patient autonomy (in line with the 2005 legislation), it also tends to be largely influenced by what the physician actually wants to do. The possibility of advance directives was introduced with the 2016 law, similar to the “living will” concept in the USA, but they are not yet common among the population.

The collegial decision-making procedure is only required when the patient is unable to express themselves. If advance directives exist, they are taken into account. If the family is available, they are asked about what the patient would have wanted (if they know). As mentioned above, the physician is supposed to inform the family about a non-readmission decision, but in practice, this doesn’t always happen. The novelty of this study is to reveal these gaps in practice. As noted by Nates et al in the guidelines for ICU Admission, Discharge, and Triage from the Society of Critical Care Medicine: “We suggest that a standardized process for discharge from the ICU be followed; both oral and written formats for the report may reduce readmission rate” [2].

Despite the methodological strengths of this multicentre study, and the large number of physicians who participated, there are nonetheless some limitations. Firstly, the questions on the interview guide may not have covered the full spectrum of dimensions related to the question of non-readmission. Second, only physicians were interviewed; nurses and/or nurses’ aides were not included, but they might have had interesting and complementary insights to provide. Thirdly, the time of day (point during the work shift) at which the interviews were performed might also have had an influence on the tone and content of the response. Fourthly, patient/family perspectives were not included in this study, and we know very little about how patients/families perceive non-readmission decisions being made by the clinical team.

## Conclusion

This qualitative study sheds light on the difficulties encountered by ICU physicians when making decisions about non-readmission of patients to the ICU. However, areas for improvement are identified, such as anticipating the possibility of ICU admission during discussions of the patient’s goals of care. It also appears that certain aspects of this type of decision could be facilitated by the regular organisation of formal meetings specifically to discuss readmission at the end of the patient’s initial ICU stay. These essential developments in the intensive care specialty will require collective engagement and multidisciplinary consultation for a more balanced decision-making process that includes the patient/family in discussions and decisions along the entire trajectory of the patient’s course.

## Supporting information

S1 Checklist Consolidated criteria for reporting qualitative studies (COREQ): 32-item checklist. (DOCX)

S1 Table Interview guide in French. (DOCX)
