# Supplementary material for: Non-readmission decisions in the intensive care unit: A qualitative study of physicians’ experience in a multicentre French study
Source: PLoS One. 2021 Jan 14;16(1):e0244919. doi: 10.1371/journal.pone.0244919 (PMC7808577; doi:10.1371/journal.pone.0244919)
Supplement: S1 Checklist — (DOCX) [file pone.0244919.s001.docx]

**Consolidated criteria for reporting qualitative studies (COREQ): 32-item checklist**

Developed from:

Tong A, Sainsbury P, Craig J. Consolidated criteria for reporting qualitative research (COREQ): a 32-item checklist for interviews and focus groups. *International Journal for Quality in Health Care*. 2007. Volume 19, Number 6: pp. 349 – 357

**YOU MUST PROVIDE A RESPONSE FOR ALL ITEMS. ENTER N/A IF NOT APPLICABLE**

| **No. Item** | **Guide questions/description** | **Reported on Page #** |
| --- | --- | --- |
| **Domain 1: Research team and reﬂexivity** |  |  |
| *Personal Characteristics* |  |  |
| 1. Inter viewer/facilitator | Which author/s conducted the interview or focus group? | The interviews performed to develop the questionnaire were performed by NMB, the study interviews were conducted by MJ and NMB. |
| 2. Credentials | What were the researcher’s credentials? E.g. PhD, MD | Sociologist for Nicolas Meunier-Beillard, and PhD, MD for Jean-Pierre Quenot and Jean-Philippe Rigaud, and MD for MJ. The highest degrees for each researcher are given on the title page. |
| 3. Occupation | What was their occupation at the time of the study? | Sociologist for Nicolas Meunier-Beillard, and physicians for Jean-Pierre Quenot and Jean-Philippe Rigaud and Marine Jacquier This is stated in the Methods section, |
| 4. Gender | Was the researcher male or female? | One male and one female, this is specified in the methods section. |
| 5. Experience and training | What experience or training did the researcher have? | The sociologist has a Masters degree in sociology, and the physicians have more than 5 years of experience in the ICU, and more than 10 years of third-level education. All had wide experience of qualitative research, as witnessed by their scientific publications (list available in Medline). This is stated in the methods.  Methods |
| *Relationship with participants* |  |  |
| 6. Relationship established | Was a relationship established prior to study commencement? | N/A . |
| 7. Participant knowledge of the interviewer | What did the participants know about the researcher? e.g. personal goals, reasons for doing the research | An information letter describing the research was given to participants. Consent to participation was assumed by the fact that participation was voluntary. |
| 8. Interviewer characteristics | What characteristics were reported about the inter viewer/facilitator? e.g. Bias, assumptions, reasons and interests in the research topic | The assumptions behind the development of questionnaire were based on empirical interviews as outlined in the Methods section. |
| **Domain 2: study design** |  |  |
| *Theoretical framework* |  |  |
| 9. Methodological orientation and Theory | What methodological orientation was stated to underpin the study? e.g. grounded theory, discourse analysis, ethnography, phenomenology, content analysis | Methods.  The discourse from the interviews was analyzed with textual content analysis |
| *Participant selection* |  |  |
| 10. Sampling | How were participants selected? e.g. purposive, convenience, consecutive, snowball  . | Methods  The participants were selected from the physicians of several intensive care units and agreement for participation was given by all physicians. They were invited to participate by personal invitation. |
| 11. Method of approach | How were participants approached? e.g. face-to-face, telephone, mail, email | In person. |
| 12. Sample size | How many participants were in the study? | Results; 22 physicians. |
| 13. Non-participation | How many people refused to participate or dropped out? Reasons? | None |
| *Setting* |  |  |
| 14. Setting of data collection | Where was the data collected? e.g. home, clinic, workplace | Data collected at the hospital; Methods |
| 15. Presence of non-participants | Was anyone else present besides the participants and researchers? | No |
| 16. Description of sample | What are the important characteristics of the sample? e.g. demographic data, date | The characteristics of the respondents are given in the Results. |
| *Data collection* |  |  |
| 17. Interview guide | Were questions, prompts, guides provided by the authors? Was it pilot tested? | The questionnaire was tested for the comprehension of each question with a panel of physicians different from those participating in the present study (Methods) |
| 18. Repeat interviews | Were repeat inter views carried out? If yes, how many? | No |
| 19. Audio/visual recording | Did the research use audio or visual recording to collect the data? | Audio recording (Methods) |
| 20. Field notes | Were ﬁeld notes made during and/or after the inter view or focus group? | No, since there were audio recordings |
| 21. Duration | What was the duration of the inter views or focus group? | The average duration of the interviews is given in the abstract and results. |
| 22. Data saturation | Was data saturation discussed? | Yes; Methods |
| 23. Transcripts returned | Were transcripts returned to participants for comment and/or correction? | No. |
| **Domain 3: analysis and ﬁndings** |  |  |
| *Data analysis* |  |  |
| 24. Number of data coders | How many data coders coded the data? | 2, with triangulation among a larger group |
| 25. Description of the coding tree | Did authors provide a description of the coding tree? | Since the interviews were confidential, this data is not freely available, but reasonably requests to the first author will be considered. |
| 26. Derivation of themes | Were themes identiﬁed in advance or derived from the data? | The themes were developed from the interviews as described in the Methods |
| 27. Software | What software, if applicable, was used to manage the data? Not applicable | NVivo |
| 28. Participant checking | Did participants provide feedback on the ﬁndings? | No |
| *Reporting* |  |  |
| 29. Quotations presented | Were participant quotations presented to illustrate the themes/ﬁndings? Was each quotation identiﬁed? e.g. participant number | Yes, citations are given to illustrate the themes. |
| 30. Data and ﬁndings consistent | Was there consistency between the data presented and the ﬁndings? | Yes (Results). |
| 31. Clarity of major themes | Were major themes clearly presented in the ﬁndings? | Yes (Results) |
| 32. Clarity of minor themes | Is there a description of diverse cases or discussion of minor themes? | Yes Discussion |

**Once you have completed this checklist, please save a copy and upload it as part of your submission. When requested to do so as part of the upload process, please select the file type: *Checklist*. You will NOT be able to proceed with submission unless the checklist has been uploaded. Please DO NOT** **include this checklist as part of the main manuscript document. It must be uploaded as a separate file.**
