# Supplementary material for: Non-readmission decisions in the intensive care unit: A qualitative study of physicians’ experience in a multicentre French study
Source: PLoS One. 2021 Jan 14;16(1):e0244919. doi: 10.1371/journal.pone.0244919 (PMC7808577; doi:10.1371/journal.pone.0244919)
Supplement: S1 Table — (DOCX) [file pone.0244919.s002.docx]

**S1 Table. Interview guide in French.**

1. A votre avis, l’admission en réanimation était-elle appropriée et/ou anticipée ?
2. Est-ce que vous demandez si des directives anticipées ont été formulées ?
3. Comment la décision de non-réadmission est-elle prise, dans votre pratique quotidienne ?
4. Quel est le profil de patients à ne pas réadmettre en réanimation ?
5. Quelle est la place d’une équipe mobile de soins palliatifs ?
6. Les décisions de non-réadmission sont-elles gravées dans le marbre ou peuvent-elles être revues, à votre avis ?
